# Deploying and Maintaining Standards of New Pharmacy Services Provision in Poland-Introducing the National Pharmacist Competencies Assessment Tool: Pre-Registration Exam–Results of the Pilot Project

**DOI:** 10.3390/ijerph19137809

**Published:** 2022-06-25

**Authors:** Piotr Merks, Urszula Religioni, Aleksandra Howell, Marvin Munzu, Edwin Panford-Quainoo, Agnieszka Neumann-Podczaska, Radosław Jaskólski, Beata Kaczmarek, Justyna Kaźmierczak, Eliza Blicharska, Paweł Olczyk, Agnieszka Barańska, Magdalena Waszyk-Nowaczyk, Jerzy Krysiński

**Affiliations:** 1 Faculty of Medicine, Collegium Medicum, Cardinal Stefan Wyszyński University, 01-815 Warsaw, Poland; piotrmerks@googlemail.com; 2 Collegium of Business Administration, Warsaw School of Economics, 02-554 Warsaw, Poland; 3 School of Public Health, Centre of Postgraduate Medical Education of Warsaw, 01-813 Warsaw, Poland; 4 Department of Pharmacy, Oxford University Hospitals NHS Foundation Trust, Oxford OX3 9DU, UK; aleksandra.howell@nhs.net; 5 Day-Lewis Pharmacy, Huntingdon PE29 3RL, UK; gphccourse@gmail.com; 6 Liverpool School of Tropical Medicine, Liverpool L3 5QA, UK; 248964@lstmed.ac.uk; 7 Department of Palliative Medicine, Poznan University of Medical Sciences, 61-245 Poznan, Poland; ar-n@wp.pl (A.N.-P.); be.kaczmarek@gmail.com (B.K.); 8 Department of Pharmaceutical Technology, Faculty of Pharmacy, Collegium Medicum in Bydgoszcz, 85-089 Bydgoszcz, Poland; radjaskolski@gmail.com (R.J.); jerzy.krysinski@cm.umk.pl (J.K.); 9 Zdrowit sp. z o.o., Pharmacy Chain, ul. Diamentowa 3, 41-940 Piekary Śląskie, Poland; j.kazmierczak@aptekizdrowit.pl; 10 Department of Analytical Chemistry, Medical University of Lublin, Chodźki 4a, 20-093 Lublin, Poland; elizablicharska@umlub.pl; 11 Department of Community Pharmacy, Faculty of Pharmaceutical Sciences, Medical University of Silesia, 40-027 Katowice, Poland; polczyk@sum.edu.pl; 12 Department of Medical Informatics and Statistics with e-Health Lab, Medical University of Lublin, 20-059 Lublin, Poland; agnieszkabaranska@umlub.pl; 13 Department of Pharmaceutical Technology, Poznan University of Medical Sciences, 61-701 Poznan, Poland; mwaszyk@ump.edu.pl

**Keywords:** pharmacist, education, pharmaceutical education, educational standards, Poland

## Abstract

Despite the functioning of the Bologna Declaration, the knowledge and skills of graduates educated in different countries may differ significantly. Therefore, this article aims to present the differences in results of the final exam in pharmacy among Polish pharmacy students. This exam was modeled on the British national exam supervised by the General Pharmaceutical Council. The exam was conducted in three cities in Poland, among a total of 175 final-year students (a full sample of those eligible was 451 with 276 refusals (38.58% response rate)). Taking the exam was voluntary and anonymous. The results indicate that none of the Polish students achieved the 70% mark required to pass the Great Britain exam. Significant differences in test results were noticed between cities. Students achieved the best average exam result in Bydgoszcz (46.35%), then in Warsaw (38.81%) and Łódź (38.35%). The pharmaceutical education system in Poland requires complete changes that will prepare future pharmacists for clinical work.

## 1. Introduction

Pharmacists are some of the primary medical professionals in the healthcare sector. The main task of pharmacists is to improve patients’ health and quality of life in the area of pharmacotherapy, regardless of whether they work in community or hospital pharmacies. The essential role of the pharmacist is to optimize patient treatment, including detection of adverse events and other drug errors. The patient counselling and significant influence of pharmacists on the increase in patients’ adherence to the therapies is also emphasized. Furthermore, given the constantly growing health care spending, pharmacists’ role in the optimization of therapy costs should be indicated [1,2,3].

The World Health Organization (WHO) and other organizations such as the American Society of Health-System Pharmacists (ASHP) emphasize that pharmacists are the health professionals most accessible to patients [4,5]. Therefore, they should be involved in patient care. The role of a pharmacist in different countries, including within Europe, varies significantly [6,7]. Many of these differences are due to curricula, including soft skills learning through pharmaceutical education. 

### 1.1. Pharmacy Education in Poland

In Poland, pharmaceutical studies are part of education in the field of medical sciences, health sciences, and physical education sciences. Studies last no shorter than 11 semesters, ending with the statutory work placement of six months. In the academic year, there are two semesters divided into 15 weeks each. Additionally, after the 3rd and 4th year of studies, students complete one month of professional practice during the summer holidays [8,9]. The number of hours of classes and internships will be at least 5300. The studies are master’s studies. The number of ECTS points (European Credit Transfer and Accumulation System) necessary to complete the studies cannot be less than 300. The education standards of future pharmacists in Poland combine general and detailed learning outcomes, including knowledge in pharmaceutical, medical, biological, chemical, and social sciences. The pharmacy students in Poland obtain thorough knowledge about drugs and substances used in their production, pharmaceutical technology, metabolism, and effects of drugs, as well as the correct use of medicinal products. Students learn the methods and techniques of researching medicinal products in terms of chemical, pharmacological and toxicological properties. They gain knowledge of the basics of pharmaceutical law and management in pharmacy, including the principles of ethics and deontology. The education process is carried out in the form of classes or groups of classes corresponding to individual issues in the discipline scientific—pharmaceutical sciences (e.g., synthesis and technology of therapeutic agents, drug chemistry, drug form technology, pharmacology, pharmacodynamics, pharmacokinetics); groups of integrated classes combining two or more issues from a scientific disciplines—pharmaceutical sciences (e.g., pharmaceutical care and social pharmacy, methods molecular biology and pharmaceutical biotechnology); and multidisciplinary groups of classes devoted to specific issues (e.g., drug design, clinical pharmacy, industrial pharmacy) [9].

Polish legal acts indicate that a graduate of pharmacy studies is required to achieve competence in the preparation, manufacture and quality assessment of medicinal products, providing reliable and objective information on the medicinal products and medical devices. A pharmacy graduate should understand the principles of pharmacotherapy rationalization, as well as skillfully conduct the research on both substances and medicinal products. General effects in the process of educating pharmacists in Poland in terms of knowledge and skills also take into account social competences by preparing future pharmacists to work in community pharmacies, hospital pharmacies, pharmaceutical wholesalers, the pharmaceutical industry, pharmaceutical inspection as well as other offices and institutions, both state and local, operating in the field of pharmacy and healthcare [9].

### 1.2. Pharmacy Education in the UK

The education of pharmacists in the UK is regulated by an independent body of the General Pharmaceutical Council (GPhC). GPhC aims to protect and promote the health, safety, and well-being of people, especially those who need the services of pharmacists. Its main tasks include setting the requirements for practicing the profession, approving pharmacists and pharmacy technicians’ qualifications, and keeping a register of pharmacists, pharmacy technicians, and pharmacies [10].

The General Pharmaceutical Council accredits universities educating students in pharmacy, ensuring pharmacists’ quality of education. The Master of Pharmacy (MPharm) studies in Great Britain last four years [10]. Students are obliged to take at least 3000 h of classes directly related to pharmaceutical subjects during their studies, especially in relation to clinical practice. During their studies, students gain, amongst others, knowledge about the effects of drugs, the functioning of the human body, and broad universal competencies, including problem-solving, clinical decision-making, effective communication, numerical data analysis [10]. Students preparing to practice as pharmacists in the United Kingdom gain practical skills from the first year of study. They are exposed to working with the patients during which students also have the possibility of practical problem-solving and pharmaceutical care planning, resulting from the educational standards set by the GPhC.

After graduation from their respective Universities, future pharmacists perform a 12-month obligatory internship in a community pharmacy, hospital pharmacy, and the industry (or combining different types of practices). Completing the training is associated with taking the independent state examination conducted by the Royal Pharmaceutical Society, and obtaining a positive grade for this examination qualifies one to practice as a pharmacist.

### 1.3. Aim of the Article

This article aims to compare the programs and standards of teaching the pharmacist profession in Poland and the United Kingdom (UK), including the final examination results, designed in Poland, similar to the pre-registration exam conducted at the end of pharmaceutical studies in the UK. The results of these considerations may be used to define new directions for pharmacists’ education in Poland, including the development of recommendations and standards for teaching this profession in Poland.

## 2. Materials and Methods

Based on the final exam after pharmaceutical studies in the UK, accredited by the General Pharmaceutical Council, we prepared the exam in Polish for Polish pharmacy students. The exam consisted of 45 test questions and four calculation questions, to be calculated without using a calculator. The test was divided into five different education modules, which had different forms of questions:

- Part I (13 questions)—simple completion answers;
- Part II (11 questions)—the best answer to the given question (multiple completion answers);
- Part III (12 questions)—the question of choosing the best most appropriate classification answers;
- Part IV (9 questions)—true/false (statement questions);
- Part V (4 questions)—calculation questions.

Sample questions are included in Appendix A. The exam is passed when the student obtains a minimum of 70% of the points.

## 3. Results

Students had 60 min to complete the test, and participation in the test was anonymous. The exam was conducted at Polish universities educating in the field of pharmacy in 2018. It was attended by fifth-year pharmacy students (volunteers) from universities in the following cities: Bydgoszcz 77 people (92.8% of all students), Warsaw 55 people (38.2%), and Łódź 43 people (39.1%). A total of 175 people took the exam (response rate 38.8%). A full sample of those eligible was 451.

The best average exam result was achieved by students from Bydgoszcz (46.35%), then from Warsaw (38.81%) and Łódź (38.35%) (Table 1).

The minimum and maximum results obtained on the test by students in individual cities are as follows: Warsaw: minimum value 12.2%, maximum 65.3%; Bydgoszcz: minimum value 20.4%, max. 65.3%; Łódź: minimum value 24.5%, maximum 53.1%. The above results indicate that none of the students taking the exam reached the 70% threshold for passing the test.

## 4. Discussion

Pharmacists are an integral part of health care systems [11,12,13]. They have the experience to detect, resolve, and prevent drug-related problems [14,15]. In many countries, such as the United Kingdom, pharmacists are involved in the interdisciplinary Primary Care Teams (PCT). They directly take care of the patient through activities such as participating in drug management and drug counselling, identifying undesirable or incorrect drugs, and managing chronic diseases [16]. These activities directly impact the improvement of health effects and the quality of life of patients [17]. 

Highly developed pharmaceutical education, which begins during studies, is a prerequisite for pharmacists’ full and beneficial involvement in patient care. Despite the fact that both Poland and Great Britain signed the Bologna Declaration’s assumptions, aimed at unifying the higher education systems of European countries, the teaching models in the discussed countries are significantly different. In the UK education programs, the clinical aspect of the classes is emphasized, which is the basis for practical preparation for broadly understanding of pharmaceutical care. Internships taking place from the first years of study train future pharmacists to collaborate with representatives of various medical professions, including doctors. This interprofessional model can significantly influence the current practice where decisions to treat the patient are discussed between doctors and pharmacists.

The role of the pharmacist in the United Kingdom is crucial [18]. While pharmacists in the UK are PCT members and take direct care of patients, in Poland, this situation does not exist.

The UK education system is widely regulated and independently supervised, including the state final examination. Notably, the state exam may have a maximum of three attempts. In the event of a triple failure on the test, students may not be allowed to work as pharmacist. The results indicating that none of the surveyed graduates of Polish universities obtained the required number of points emphasize the relatively low level of pharmacy education. Data obtained from the General Pharmaceutical Council show that, in 2019, the average pass rate in the United Kingdom is 72.33%.

This will be a good point to also outline the pharmacy education system in Poland to compare and contrast the sections above. For this reason, it is necessary to develop a new model of education in Poland, based on the International Pharmaceutical Federation (FIP) guidelines and competency-based pharmacy education (CBPE) [19]. In order to assess the knowledge and competencies of future pharmacists, it is recommended to introduce a final exam at Polish universities, which would be supervised by an independent organization. The real threat to these activities may turn out to be a noticeable downward trend in the number of new pharmacists, which already amounts to 1.85 pharmacists per pharmacy and is much lower than the number of pharmacists per pharmacy in other OECD countries (2.40) [20].

## 5. Conclusions

The pharmaceutical education system in Poland requires complete changes. However, these modifications seem necessary to increase the pharmacist’s role in the health care system and ensure the complete safety of patients. Although pharmacists are often overlooked in setting primary health goals, and their role in promoting health in many countries is still low, it should be noted that pharmacists are the medical profession most frequently visited by patients. Thus, great emphasis should be placed on educating pharmacists who can actively support patients’ health in the area of pharmacotherapy, being also health educators.

### Limitations

One of the main study limitations was the number of universities with faculty of pharmacy who are engaged in the project. The information was distributed to all of them, via the student organisations Polish Pharmaceutical Student Association (PTSF) organisation, which was supporting the project. The official letters were directly sent to the dean of the faculty of pharmacy for consent. Only three universities responded positively and decided to take on the challenge of the project.

It should be noted that, despite the general framework for educating pharmacy students in Poland, programs between universities may differ. Thus, students in other cities (not included in the study) could have had other results of this exam.

Additionally, in order to get a complete picture of the education of students, a pre-assessment should be carried out. In our study, we only show the results of the final exam. However, it seems to us that further research, taking into account pre-assessment, would significantly enrich the research area.

In the future, we would like to plan another similar project with a larger national scope and range. The Polish pharmacy student education framework does not focus on clinical skills, hence the results show that the calculation part was passed on the higher level. Students achieve the lowest level in the area of clinical skills. Currently, all universities are interpreting pharmaceutical care in a very personal way, and the level of education varies from one university to another, which can also create a potential bias. Moreover, very often, a random perception and overestimation of teachers skills who are fully theoretical can result in not meeting certain education goals due to a lack of practical clinical skills and knowledge. Clinical pharmacy does not exist at the moment in Poland. There is no clinical intervention in the community pharmacy at the moment.

We believe that there should be a national standardisation centre for pharmaceutical care and pharmacist competency framework education which could develop a standard that will support the global stream for pharmacy development in the country maintaining the same level in every pharmacy school in every region of the country. At the time when this pilot was prepared, there were no enhanced and advanced services available in Poland. However, there is still a problem with standardisation in education, and it is now a subject for national discussion, after vaccination against COVID 19 and Flu were introduced; in addition, a national medicine use review pilot is about to start. Our plan in the future is to build an alliance with international organisations, which could help us to build a new pharmacist competency framework in Polish pharmacy schools. This paper and its research were created purely for scientific reasons, to show the national necessity of a significant need to change the pharmacy education and rebuild the pharmacist competency framework to a more practical and clinical role.

## Figures and Tables

**Table 1:** Average exam results in particular cities.

	Part I	Part II	Part III	Part IV	Part V	Overall
Warsaw	49.51%	37.69%	41.52%	25.66%	28.64%	38.81%
Bydgoszcz	62.44%	28.22%	48.16%	30.16%	74.35%	46.35%
Łódź	43.83%	38.05%	38.37%	33.85%	31.40%	38.35%

## Data Availability

All data are available from the corresponding author.

## Abbreviations

ASHP	American Society of Health-System Pharmacists
CBPE	competency-based pharmacy education
FIP	International Pharmaceutical Federation
GPhC	General Pharmaceutical Council
MPharm	Master of Pharmacy
OECD	Organisation for Economic Cooperation and Developmen
PCT	Primary Care Teams
UK	United Kingdom
WHO	World Health Organization.

## Appendix A

Sample Questions included in the Exam

**Part I** (13 questions)—simple completion answers

for example:

What time of the day are steroids taken?

(a) in the morning

(b) at noon

(c) in the evening

(d) at bedtime

**Part II** (11 questions)—the best answer to a given question (multiple completion answers)

for example:

1. causes hypothyroidism?

2. causes hypokalemia?

3. is an enzyme activator?

A lit

B rifampicin

C amiloride

D clarithromycin

E ciprofloxacin

**Part III** (12 questions)—the question of choosing the best most appropriate classification answers

for example:

Which set does the interaction occur with?

1. kaptopril + atenolol

2. propranolol + amlodipine

3. Ibuprofen + furosemide

(a) 1, 2, 3

(b) 1, 2

(c) 2, 3

(d) 1

(e) 3

**Part IV** (9 questions)—true/false (statement questions)

for example:

First claim:

Metformin is the drug of the first choice in the treatment of type II diabetes mellitus with obesity.

Second claim:

The side effect of metformin is anorexia.

**Table A1:** Possible answers.

	The First Claim	The Second Claim	
A	Truth	Truth	The second claim correctly explains the first one
B	Truth	Truth	The second claim does NOT correctly explain the first one
C	Truth	False	
D	False	Truth	
E	False	False	

**Part V** (4 questions)—calculation questions

for example:

What volume of water is needed to dissolve 150 mg of the substance obtaining a 5% *w*/*v* liquid?

(a) 20 mL

(b) 30 mL

(c) 2 mL

(d) 3 mL

(e) 5 mL
